# Development of a Cell-Based Assay for Measuring Base Excision Repair Responses

**DOI:** 10.1038/s41598-017-12963-7

**Published:** 2017-10-11

**Authors:** Tyler Golato, Boris Brenerman, Daniel R. McNeill, Jianfeng Li, Robert W. Sobol, David M. Wilson

**Affiliations:** 1 Laboratory of Molecular Gerontology, National Institute on Aging, Intramural Research Program, National Institutes of Health, 251 Bayview Blvd., Ste. 100, Baltimore, MD 21224 USA; 2 Molecular and Metabolic Oncology Program, USA Mitchell Cancer Institute, University of South Alabama, 1660 Springhill Avenue, Mobile, AL 36604 USA

## Abstract

Base excision repair (BER) is the predominant pathway for coping with most forms of hydrolytic, oxidative or alkylative DNA damage. Measuring BER capacity in living cells is valuable for both basic science applications and epidemiological studies, since deficiencies in this pathway have been associated with cancer susceptibility and other adverse health outcomes. At present, there is an ongoing effort to develop methods to effectively quantify the rate of BER as a whole. We present a variation of a previously described “Oligonucleotide Retrieval Assay” designed to measure DNA excision repair that is capable of quantifying the rate of repair of thymine glycol in a variety of human cells with a high degree of sensitivity.

## Introduction

Base excision repair (BER) is a biochemical pathway that defends the human genome from many common forms of DNA damage^1^. Major substrates of BER include DNA modifications that arise as products of natural hydrolytic decay (e.g., uracil) or from reactions with endogenous chemicals, primarily reactive oxygen species created during mitochondrial oxidative phosphorylation. If unrepaired, DNA damage has the capacity to result in (i) permanent genetic change, either by error-prone replication or unwanted recombinational events, or (ii) activation of cell death responses upon arrest of a DNA or RNA polymerase. These cellular outcomes are the backdrop for carcinogenesis and degenerative pathologies, and consistently, defects in BER lead to developmental failings, cancer predisposition, immunodeficiency and neurological disease.

The BER pathway consists of five major biochemical steps: 1. Excision, where a damaged base is detached from the DNA backbone by one of several substrate-selective DNA glycosylases, such as human endonuclease III (NTH1L) that removes thymine glycol (TG)^2^. 2. Incision, where the phosphodiester backbone is cut at the resulting abasic site by an apurinic/apyrimidinic (AP) endonuclease, such as human APE1^3^. 3. End-cleanup, where the termini of the DNA break are edited to be suitable for ligation and/or extension by a DNA polymerase; this step can engage a collection of proteins, such as DNA polymerase β (POLβ), APE1, tyrosyl-DNA phosphodiesterase 1 (TDP1), aprataxin (APTX) or polynucleotide kinase 3′-phosphatase (PNKP)^4^. 4. Gap Filling, where one of a handful of DNA polymerases, such as POLβ, POLɛ or POLδ, can carry out DNA synthesis, incorporating one or several nucleotides by using the undamaged strand as a template^5^. 5. Ligation, where DNA ligase 3, nuclear isoform α (LIG3α), typically in complex with X-ray cross-complementing protein 1 (XRCC1), or DNA ligase 1 (LIG1) seals the phosphodiester backbone to complete the repair reaction^6,7^. Notably, BER can proceed via either a single-nucleotide (short-patch) or long-patch (2 to 10 nucleotides) synthesis reaction, where the latter involves strand-displacement by a PCNA-dependent replicative polymerase, such as POLɛ or POLδ, and the 5′-flap structure-specific endonuclease, FEN1. The factors determining the choice between short- and long-patch repair are not fully understood, but include the type of lesion, cell cycle stage, and whether the cell is actively dividing^8^.

A handful of mostly unrelated genetic disorders arise from inherited mutations in genes associated with BER^1^. For example, pathogenic mutations in the MutY homolog (*MUTYH*) and *NTH1* (a.k.a., *NTHL1*) DNA glycosylases have been linked to colorectal cancer^9,10^, while mutations in the uracil DNA glycosylase (*UNG*) have been associated with the immunodeficiency disorder, hyper-IgM syndrome^11^. In addition, the rare genetic neurological disorders, spinocerebellar ataxia with axonal neuropathy 1 (SCAN1)^12^ and ataxia with oculomotor apraxia 1 (AOA1)^13^, stem from mutations in genes involved in termini clean-up as part of single-strand break repair (*TDP1* and *APTX*, respectively). Thus, evidence indicates that, at least in certain circumstances, significant functional defects in BER or SSBR can give rise to human disease, presumably by either leading to mutagenesis or cell death due to the accumulation of unrepaired DNA damage.

Notably, homozygous deletion of several of the central components of BER leads to embryonic or post-natal lethality in mice, suggesting that null (or near-null) phenotypes in BER will be uncommon (at best) in the population^1^. However, studies examining the consequences of deficiencies in BER (e.g., haploinsufficiency) have been conducted in model organisms, namely mice, and reveal that partial defects in BER can increase disease susceptibility^14^. In fact, it is likely that a deficiency in BER would enhance the health risk of an organism to external stress; so while the impaired pathway may be sufficient to handle the day-to-day wear and tear of normal oxidation, stressful conditions (e.g., environmental exposure or situational events) will generate DNA damage that exceeds the organism’s intrinsic repair capacity. For instance, vulnerability to adverse conditions, such as injuries involving ischemia/reperfusion, appears to be elevated when BER is not fully operational^15–20^.

At present, BER efficiency has been difficult to measure in a holistic way in cell samples, especially human material. The current techniques for quantitation of BER fall into four major categories: (i) extract-based *in vitro* single step techniques; (ii) *in vivo* single step techniques, mainly consisting of molecular beacon substrates; (iii) the host cell reactivation (HCR) assay; and (iii) the Comet assay^1^. Other techniques exist, but they are generally less popular, primarily due to the need for complex instrumentation. Extract-based techniques typically measure the activity of an individual BER enzyme, and have primarily focused on APE1 incision^21,22^. Their advantages are the high degree of precision (implementation of a defined synthetic DNA substrate) and a reasonable degree of reproducibility, but their relevance to patient studies is hampered by a variety of factors. For example, single-step assays may miss coordination defects within the pathway, such as pathway imbalances, or fail to capture deficiencies in non-enzymatic components, such as XRCC1, which is a scaffold protein. Molecular beacon substrates have been applied to *in vitro* extract-based assays, as well as cell-based analysis following transfection^23,24^. However, similar limitations apply to the *in vivo* molecular beacon strategies, as only a single enzymatic step is being assessed.

Presently, there are only a small number of techniques that can measure the rate of DNA repair pathways *in vivo*, most notable among these are the HCR and Comet assays. The Comet assay is a powerful single-cell electrophoresis technique that can measure overall DNA damage within the nuclear genome^25^. However, the method is very sensitive to day-to-day variability, and thus, it is difficult to achieve consistent reproducibility. Furthermore, the assay requires careful experimental design to disentangle BER activity from other cellular processes that can modify DNA damage levels detected by this technique (e.g., antioxidant defenses). HCR is another strategy for measuring DNA repair *in vivo* and employs a defined DNA plasmid that is introduced into a target cell population^26^. Unfortunately, HCR is imperfect in that it involves a non-genomic DNA substrate and depends on the synthesis of a protein as a reporter for successful DNA repair, and thus, any alteration in protein production, independent of BER capacity, can alter the readout. Another method, ligation-mediated (LM)-PCR, while somewhat cumbersome, can be applied to any genomic locus in a cell, taking into account various cellular, chromatin or transcription contexts, and may be best utilized as a reference method to compare with less intensive approaches such as fluorescent oligonucleotides, or that described herein^27^. In light of the above limitations with the current methods, we set out to develop an alternative pathway assay for assessing BER, and describe herein the adaptation of a method (termed the oligonucleotide retrieval assay, ORA) previously reported to quantify nucleotide excision repair (NER)^26^. Our studies reveal that while the BER ORA can consistently measure repair activity in a number of human cell lines, it has features that must be taken into consideration when applying the method to a particular sample set.

## Results

### Overview of the Assay

**Figure 1:**
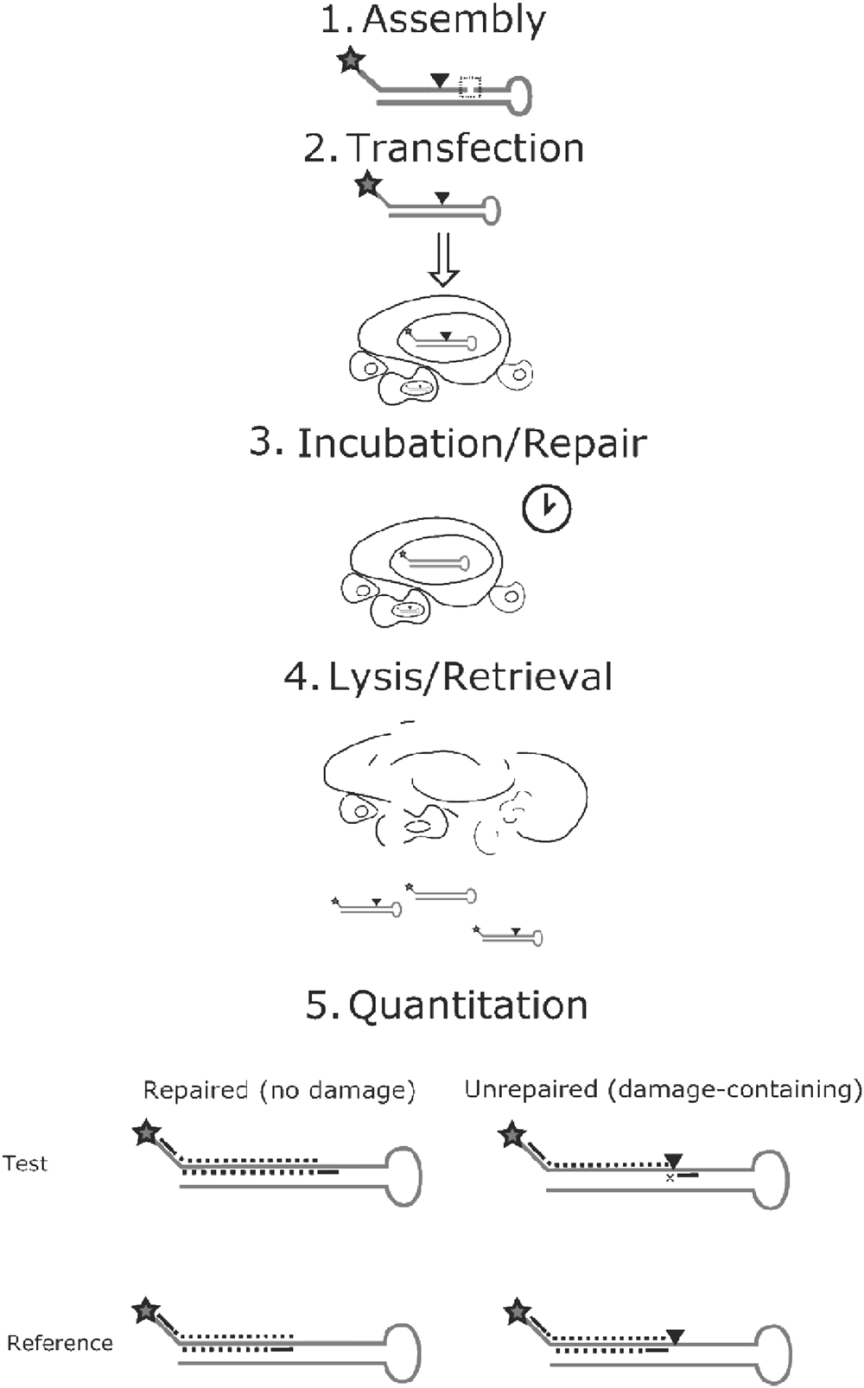
Overview of the BER ORA. The BER assay relies on assembling a complete hairpin containing a DNA lesion (upside down triangle) from smaller, easier to synthesize components, by ligation (indicated by box) in step (**1**). After ligation, the hairpin substrate is transected into the target cell population by means of chemical reagents or nucleofection in step (**2**). Cells are allowed to process the damage-containing hairpin, removing the lesion and replacing it with a normal DNA base in step (**3**). Cells are lysed with detergents or a similar method in step (**4**). qPCR detects the presence of the lesion by comparing the rate of amplification across a Test region (top) relative to a Reference region (bottom) as shown in step (**5**). Depending on whether the damage lesion is present or absent, amplification in Test will be less than (polymerase block, x) or more equal to the Reference. Star = biotin.

The cell-based BER capacity assay pursued here was founded on work described by the Loeb laboratory, who aimed to measure NER, a pathway that copes with bulky, helix-distorting DNA lesions, such as the thymine-thymine UV photodimer (TT-dimer)^28^. The general strategy of the ORA involves a 5′-biotinylated, “hairpin” oligonucleotide substrate and the following experimental steps (see general outline in Fig. 1): 1. assembly of the damage-specific DNA hairpin substrate; 2. transfection of the prepared substrate into a target cell population; 3. incubation of the transfected cells for some length of time to permit DNA repair; 4. lysis of the cells and retrieval of the biotinylated DNA from the extract; and 5. quantitative PCR (qPCR) across both the DNA damage (Test) and another defined location within the substrate (Reference). The basic principle is that if the damage (or a repair intermediate) remains, PCR amplification across this site is less efficient than if the damage had been removed and repair completed. This approach assumes the ability of the DNA lesion to block/stall a PCR polymerase. PCR of a control region on the substrate allows one to normalize to the amount of DNA retrieved, since there will likely be differences in capture efficiency from sample to sample. Thus, Test amplification relative to Reference amplification (i.e., ΔCt = Ct_Test_ − Ct_Ref_) will provide an indication of DNA repair capacity, as described in further detail below.

### Identification of a Suitable BER Lesion

**Figure 2:**
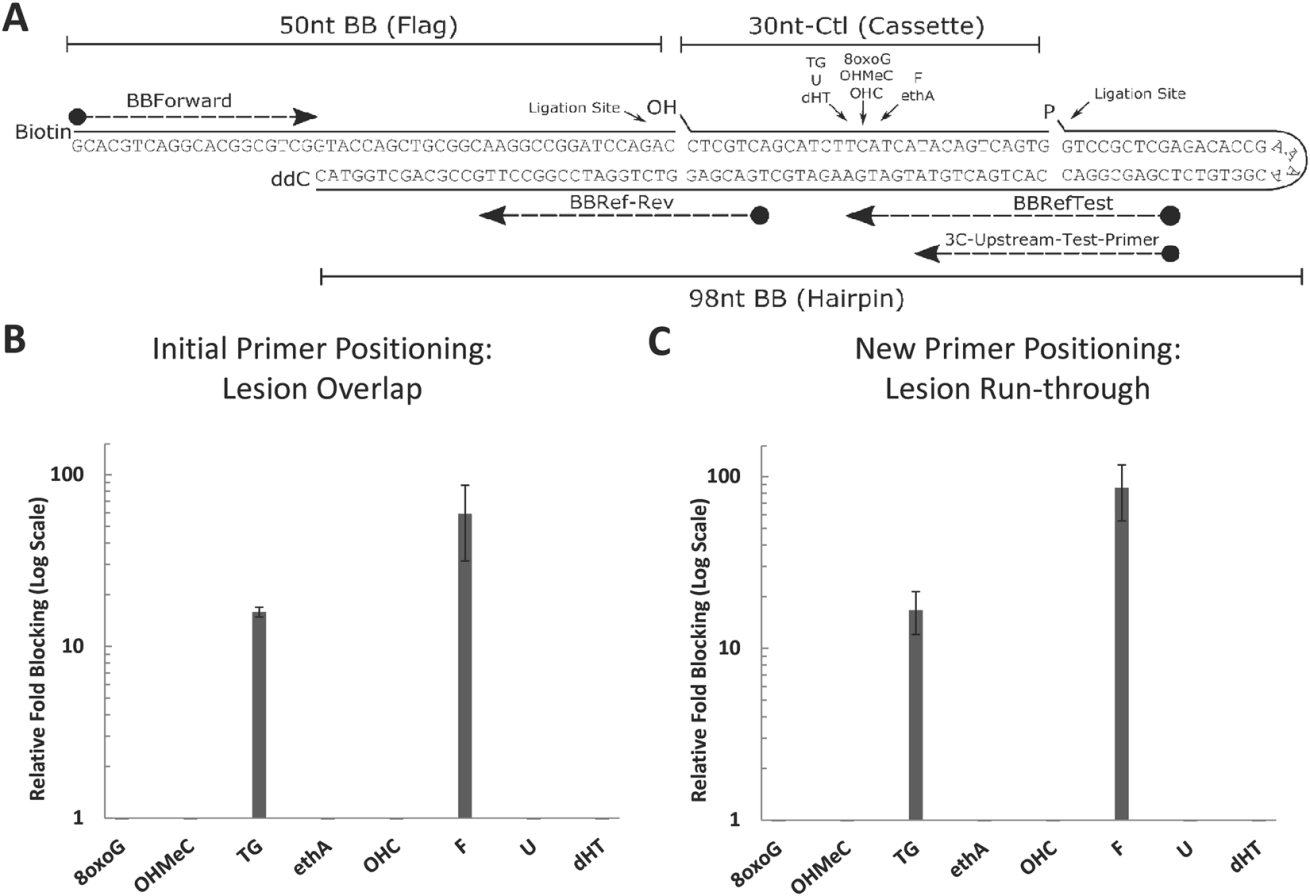
Hairpin DNA ORA Substrate and Blocking Potential of BER Damages. (**A**) The initial design of the hairpin was adapted from Shen *et al*.^28^, and contained three-components, here named Flag, Cassette, and Hairpin. The notable features of the three-component hairpin were biotinylation of the Flag oligo, a damage lesion (designated; but no 5′ phosphorylation in each of the eight different Cassette oligos), and a phosphorylation (P) and protective ddC group in the Hairpin oligo. Also shown is the positioning of the various damage sites in the three-component substrate, and the location of the qPCR primers. See Tables 1 and 2 for oligonucleotide information. (**B**) The blocking-potential of each lesion with a primer overlapping the lesion (BBRefTest). (**C**) The blocking-potential of each lesion with a primer that permits run-through (3C-Upstream-Test-Primer). Averages and standard deviations of at least three independent experimental replicates are shown in panels B and C.

Based on the design of the Loeb group^28^, our initial BER substrate consisted of three-components (Table 1): component 1 (referred to as the “flag”) harbored a 5′-biotin residue; component 2 (referred to as the “cassette”) possessed a defined, site-specific DNA damage (unless the undamaged control); and component 3 (referred to as the “hairpin”) provided the hairpin body, as well as a 3′-blocking group to prevent exonuclease degradation (depicted in Fig. 2A). Since the current ORA relies on the ability of the DNA lesion to interfere with PCR (e.g., the TT-dimer used in the original design), it was necessary to identify a suitable blocking damage. Thus, we first determined the effectiveness of several classic BER substrates to impair a standard qPCR. The damage-containing hairpin DNA substrates, which were prepared with distinct oligonucleotide cassettes (Table 1), harbored one of the following DNA modifications, all of which were commercially available for oligonucleotide synthesis: TG, 8-oxoguanine (8oxoG), hydroxymethylcytosine (OHMeC), ethenoadenine (ethA), hydroxycytosine (OHC), uracil (U), dihydrothymine (dHT) or an abasic site analog (F).

**Table 1:** Oligonucleotides for Hairpin Substrates.

Oligonucleotide Name	Sequence
*Three-component Oligonucleotides*	
50nt BB (Flag)	(biotin)GCACGTCAGGCACGGCGTCGGTACCAGCTGCGGCAAGGCCGGATCCAGAC
30nt-Ctl (Cassette)	CTCGTCAGCATCTTCATCATACAGTCAGTG (note: 5′ end not phosphorylated in three-component cassettes)
98nt BB (Hairpin)	(phosphate)GTCCGCTCGAGACACCGAAAACGGTGTCTCGAGCGGACCACTGACTGTATGATGAAGATGCTGACGAGGTCTGGATCCGGCCTTGCCGCAGCTGGTAC(ddC)
*Damage-Containing Three-component Cassettes (X = lesion position)*	
30nt-8oxoG (8-oxoguanine)	CTCGTCAGCATCTXCATCATACAGTCAGTG
30nt-16F (abasic site analog, tetrahydrofuran)	CTCGTCAGCATCTTCXTCATACAGTCAGTG
30nt-14TG (thymine glycol)	CTCGTCAGCATCTXCATCATACAGTCAGTG
30nt-16ethA (ethenoadenine)	CTCGTCAGCATCTTCXTCATACAGTCAGTG
30nt-15OHMeC (hydroxymethylcytosine)	CTCGTCAGCATCTTXATCATACAGTCAGTG
30nt-15OHC (hydroxycytosine)	CTCGTCAGCATCTTXATCATACAGTCAGTG
30nt-14U (uracil)	CTCGTCAGCATCTXCATCATACAGTCAGTG
*Two-component Oligonucleotides*	
NHD-BiotTG-Cassette	(biotin)ATTAGTGGCCTGCAGACTAGGACCACTCGGGGTAGTCGTTGGGCTTATGCACCGTAAAGTTATGGTAGTCTGACTCAGTTGGAGCTGAAT(TG)CTCCGTTATAT
NHD-HairpinBody	(phosphate)CGTGTCACGTGATGCTACGTAAAAATACGTAGCATCACGTGACACGATATAACGGAGAATTCAGCTCCAACTGAGTCAGACTACCATA(ddC *or* FAM)
TT-Dimer	(biotin)ATTAGTGGCCTGCAGACTAGGACCACTCGGGGTAGTCGTTGGGCTTATGCACCGTAAAGTTATGGTAGTCTGACTCAGTTGGAGCTGAAT(T*)CTCCGTTATAT

To determine the blocking potential of each lesion on a PCR polymerase, we utilized the quantitative strategy outlined previously^28^. We first calculated the ΔCt for the control hairpin (ΔCt_Control-HP_ = Ct_Test_ − Ct_Ref_). Subsequently, we calculated the ΔCt of the damage-containing hairpin (ΔCt_Damage-HP_ = Ct_Test_ − Ct_Ref_). Next, we determined the differential between these two measurements (ΔΔCt = ΔCt_Damage-HP_ − ΔCt_Control-HP_). The blocking potential of the lesion is calculated as a function of the fold difference between the respective ΔCt values for both reactions (2^ΔΔCt^). Percent repair can be determined by ((2^−ΔΔCt^)*100).

Figure 2A shows the position of the Test primer (BBRef Test; Table 2) relative to the different DNA lesions within the template for the initial qPCRs. As shown in Fig. 2B (see also Fig. S1), in this arrangement, only TG and F profoundly interfered with PCR amplification relative to the undamaged control template. These results are consistent with prior studies showing that AP sites and TG lesions effectively hinder DNA polymerase progression, whereas U and 8oxoG, for example, mostly do not^29,30^. To further determine the blocking potential of the different damages within the context of our three-component substrate, we positioned the Test primer (3C-Upstream-Test-Primer; Table 2) downstream of the modification to allow for polymerase “run-through” (Fig. 2A). These studies again revealed that the AP site analog (F) is the most potent blocking lesion (nearly 100-fold), while TG consistently resulted in a significant inhibition (~17-fold) of the qPCR (Fig. 2C).

**Table 2:** qPCR Primers. * = LNA.

*Three-component primers*	
BBRef-Rev	TGACGAGGTCTGGATCCGGCCTTG
BBRefTest	TCGAGCGGACCACTGACTGTATGATGA
3C-Upstream-Test-Primer	TCGAGCGGACCACTGACTGTA
BBForward	GCACGTCAGGCACGGCGTCG
*Two-component primers*	
NHD-NFwdPr1	GGCCTGCAGACTAGGA
Forward Primer 2	AGACTAGGACCACTCGG
NHD-NTestPr0	ACGTGACACGATATAACGGA
NHD-NTestPr1	CGTGACACGATATAACGGAG
NHD-NTestPr2	CGTGACACGATATAACGGAGA
NHD-NTestPr3	GTGACACGATATAACGGAGAA
NewHairpinRef3	CCAACTGAGTCAGACTACCA
NHD1LNATest	GTGACACGATATAACGGAGAA*
NHD2LNATest	GTGACACGATATAACGGAGA*A*

### Optimization of the Substrate and qPCR Strategy

**Figure 3:**
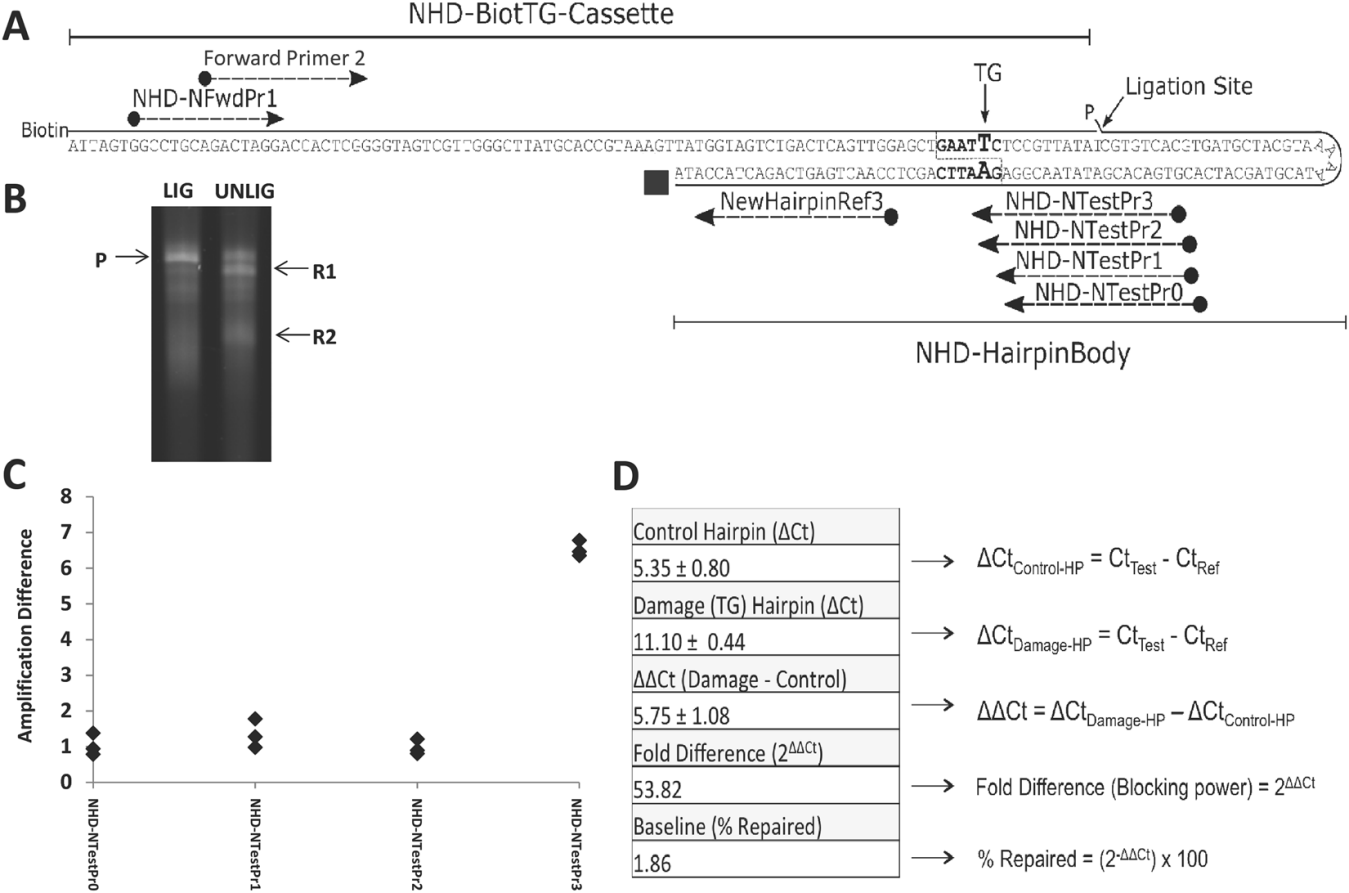
Hairpin Redesign, Ligation Optimization and Primer Positional-Dependence. (**A**) The redesigned two-component substrate eliminated the need for the kinase reaction and two ligation events, and added a restriction site around the lesion. Only one cassette containing a TG was synthesized for evaluation of this design (see Table 1). Notable features of the substrate are the biotinylation of the cassette oligo, the phosphorylation of the hairpin oligo, and the presence of a ddC or FAM (black square) acting as a blocking group. The position of the different qPCR primers is also shown (see Table 2). (**B**) Gel image showing ligated (P) and unligated reactants (R1 and R2). (**C**) Amplification differences as a function of primer position and lesion-overlap. Numbers correspond to NHD-TestPr0–3 (designated), as shown in panel A. Three independent replicates for each Test primer used are plotted. (**D**) Table describing quantitative calibration of assay, including blocking-potential of TG lesion and baseline limit of detection. HP = hairpin; control = undamaged template; damage = TG.

Since the aim of the study was to determine whether the ORA could be employed to assess the BER pathway as a whole, the remainder of the optimization involved the blocking damage TG. TG is a primary substrate of the DNA glycosylase NTH1, therefore requiring each step of the BER process to complete repair. First, to simplify and improve the efficiency of substrate creation (namely the ligation step), we converted the three-component system to a two-component design. In this new set-up (Fig. 3A), component 1 (NHD-BiotTG-Cassette) harbored both the 5′-biotin residue and the TG damage, while component 2 (NHD-HairpinBody) provided the hairpin body and the 3′-block (ddC or FAM), and was re-designed to include a 5′-phosphate to eliminate the T4 DNA kinase step. We recommend the ddC terminus going forward, as the fluorescent property of the FAM was found to interfere with the qPCR if not sufficiently diluted.

In brief, to create the BER ORA substrate, the two oligonucleotides (Table 1) were mixed at a ratio of 1:1 and incubated with T4 DNA ligase in the presence of 1 mM ATP and 10 mM DTT to seal the nick. Denaturing gel electrophoresis indicated effective ligation of the two-component system, with nearly all material migrating as fully ligated substrate (Fig. 3B, P). Second, to optimize the PCR step of the ORA, and in particular, the differential amplification across the TG residue relative to a repaired (or undamaged control) template, we examined the effect of primer positioning, temperature regimes, locked nucleic acids (LNAs), and proofreading polymerases.

#### Primer Position

In our analyses above using the three-component substrate, we found that TG was a relatively strong block to PCR amplification in two different Test primer scenarios. To further explore the effect of the location of the Test primer with respect to the TG damage in our two-component design, we examined additional oligonucleotide positions (Fig. 3C). This analysis revealed that when the primer was located such that its 3′-terminus overlapped TG by +1 nucleotide (NHD-NTestPr3), qPCR was most strongly impaired. Specifically, relative to the undamaged control template, the reaction efficiency was inhibited by up to 54-fold on the TG-containing two-component substrate using NHD-NTestPr3 (Fig. 3D). Greater polymerase-blocking efficiency ultimately translates to a more sensitive level of repair detection, and is necessary for measuring particularly low levels of repair. In light of our results, it might be interesting in the future to examine in greater depth the effects of Test primer position on the qPCRs involving the other BER lesions (see above). We point out that our qPCR approach is in contrast to the one employed for the TT-dimer NER substrate^28^, where the Test primer was located several nucleotides downstream of the damage, since this lesion is an absolute block to DNA polymerase progression.

#### PCR Conditions

Since primer binding and sequence specificity can be influenced by reaction condition temperatures, we next examined the effect of employing a touchdown qPCR strategy. Such methods are commonly used to reduce non-specific PCR amplification^31^, which can skew subsequent amplification steps. Using touchdown in qPCRs involving the optimized two-component TG substrate and the well-positioned primer (NHD-NTestPr3), we observed a further reduction in TG-specific amplification to 6.8+/−0.36 cycles, translating to an additional 2.6-fold increase in blocking power relative to the undamaged control (Fig. S2A). Since this technique doesn’t have significant downsides, we adopted it as part of the basic experimental strategy.

#### Effect of LNA

LNAs are a modified form of synthetic DNA that induce a conformational “lock” of the DNA backbone, creating primers with a greater degree of specificity. They have been used in the detection of single nucleotide polymorphisms by means of PCR^32^, suggesting that they may be useful for detecting bulky or mis-coding DNA lesions. The use of two LNAs at the 3′ end of the primer with optimal positioning for the two-component TG substrate resulted in a poisoned reaction, with no or low product formation. Conversely, the use of one LNA at the +1 position yielded an 8-fold improvement in terms of sensitivity to the presence of repaired DNA, resulting in a reaction difference of 10+/−0.8 cycles relative to the undamaged control with the LNA primer (Fig. S2B). However, NHD1LNATest (Table 2) resulted in a significantly reduced rate of product formation overall, and due to this tradeoff, we chose not to include LNAs in the qPCR primers.

#### Proofreading Polymerases

We hypothesized that proofreading polymerases would be less likely to bind to poorly matching primers, potentially giving rise to an even greater differential between PCR of a damage-containing template relative to an undamaged (repaired) control. However, we found that using proofreading polymerases (i.e., PfuUltra from Agilent, Q5 from New England Biolabs or Pfx from Life Technologies) actually decreased the performance of the qPCR assay, resulting in a differential amplification of damaged (two-component TG substrate) to undamaged template of only ~1 cycle (data not shown). One possible explanation for the reduced performance may be that proofreading polymerases can degrade the overlapping primer, generating a primer-template intermediate that is now more efficiently amplified by the polymerase via run-through synthesis.

### Application of the BER ORA to Human Cells

**Figure 4:**
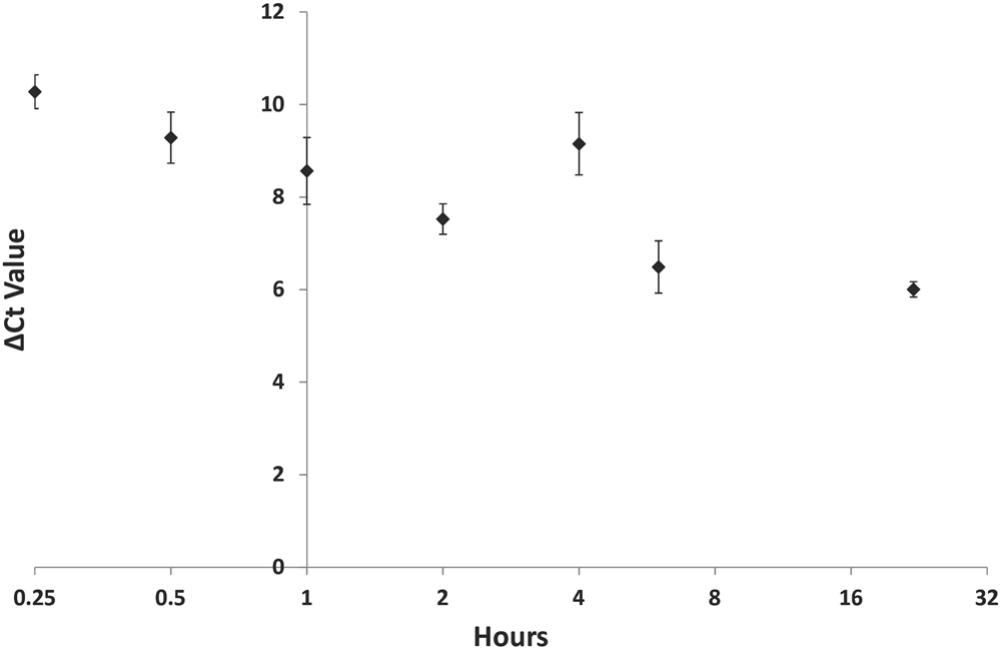
Time-dependent Repair in HEK 293T. Depiction of a time-dependent decline in ΔCt_Test_ (inversely correlated to double-strand product formation and amplification efficiency) in 293T cells after transfection with Lipofectamine 2000. Approximately 6 million cells were split into 12 wells, transfected with 800 pmol of the hairpin DNA, incubated for the indicated time periods (15 min, 30 min, 1 hr, 2 hr, 4 hr, 6 hr, and 24 hr; X-axis), and then lysed and analyzed. Decreasing ΔCt_Test_ (Y-axis) indicates the proportion of recovered repaired template is increasing. Shown is a represented analysis, as well as the averages and standard deviations of three independent experimental replicates.

Before proceeding to human cell work, we verified the assay further by examining the Reference and Test PCR products by agarose gel electrophoresis (Fig. S3A) and melting curve analysis (Fig. S3B). Both procedures showed a single major PCR product band, validating our substrate, primer pairs and qPCR conditions. Thus, with optimization of the basic methodology in place, the two-component TG-containing hairpin DNA was transfected into human embryonic kidney (HEK) 293T cells, alongside the undamaged control substrate, to quantify *in vivo* repair. We selected 293T cells for the initial work, since they were previously reported to have robust NER activity^28^. Following (i) transfection of the TG-containing or undamaged hairpin DNA into 293T cells using Lipofectamine 2000, (ii) repair incubation for up to 24 hr, and (iii) retrieval using a magnetic streptavidin-bead capture strategy, our initial analysis revealed a pattern of decreasing ΔCt_Test_ in a time-dependent fashion, indicating that repair of the transfected substrate was taking place (Fig. 4). Since repair of TG was consistently detected in 293T cells, this cell line was chosen for further optimization of the cell-based assay.

#### Transfection

Using 293T cells, we next compared several transfection methods, including four different chemical transfection reagents (i.e., Lipofectamine 2000, PolyPlus INTERFERin, Xtreme Gene HP, and Lipofectamine RNAiMax) and Amaxa Nucleofection. Due to the disparate rates of transfection and high stability of the hairpin *in vivo* (as can be concluded based on the efficient retrieval at 48 hr; unpublished observation), cells were allowed to carry out repair for 24 hr post-transfection for the comparative analysis. As shown in Fig. S4, the best results were consistently achieved with the PolyPlus INTERFERin transfection reagent. Amaxa Nucleofection and Lipofectamine RNAiMax routinely failed to show significant repair, but this was likely due to the high apparent rate of toxicity to 293T cells.

#### Lysis and DNA Retrieval

To generate cell lysates for DNA retrieval, we choose an approach that employed Qiagen Miniprep buffers (P1, P2 and P3) commonly used for plasmid DNA isolation. We reasoned that plasmid isolation techniques, such as the MiniPrep procedure, would be optimal for selectively isolating the biotinylated hairpin substrate, since such protocols allow for efficient precipitation of genomic DNA, as well as protein, enriching for the hairpin substrate with minimal contamination in the lysate. In the case of weakly adherent cells, such as HEK 293T, since initial washes with 1x PBS were prone to detaching cells, thereby decreasing cell number and yield, we harvested cells into 15 ml conical tubes before conducting the wash and lysis steps. In situations of strongly adherent cells, for example, HeLa and LN428 cell lines (see later), lysis was carried out directly in the wells where transfection had originally taken place. Since the method worked well for us in the different scenarios, only limited investigations were carried out to examine the usefulness of alternative lysis strategies, such as freeze-thaw, which was not found to improve the reliability or robustness of the assay, and often resulted in poorer performance (unpublished observation).

We next performed a comparative analysis of two different retrieval techniques: streptavidin-bead capture (Fig. S5A) and molecular exclusion filtration (Fig. S5B). Molecular exclusion columns are less laborious and allow for purification to be performed relatively quickly, and could be more suitable to high-throughput designs. After transfection of the TG-containing or undamaged hairpin DNA into 293T cells via PolyPlus INTERFERin, repair was quantified at 0, 3, 6, 9 and 12 hr following enrichment of the DNAs via one of the two strategies. This analysis revealed that the streptavidin-capture method worked most reliably for recovering the hairpin DNA, with less variability between experiments and replicates, allowing for more consistent results (unpublished observation). While molecular exclusion filtration revealed time-dependent BER, often even to a greater degree, the method was more prone to yielding variable repair measurements, and on many occasions, amplification of the recovered template failed, seemingly due to contaminants intrinsic to this form of retrieval. Thus, streptavidin-capture provided a more reliable way of ensuring that only the biotinylated material is captured, while intermediates and fragments, which may bias or interfere with repair measurements (qPCR amplification), are avoided. Though we encountered difficulties in reliably quantifying the number of molecules recovered per cell, several attempts at absolute quantification yielded results in the range of 10–500 molecules per cell, depending on the experiment and cell line. Given the similarities in the methodology employed, we estimate that our retrieval efficiency using the streptavidin-capture method is within the range observed by Shen *et al*.^28^.

#### Additional Cell Lines

**Figure 5:**
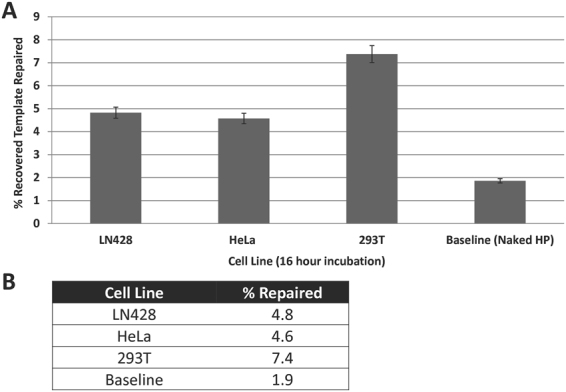
Repair Capacity in Multiple Cell Lines. Baseline for detection of repair is a function of the blocking potential of TG and is shown as “Naked Hairpin (HP)”. Using the standard protocol, BER capacity (percent of recovered template repaired) is shown for HEK 293T, HeLa, and LN428 cell lines at 16 hr. Plotted are the averages and standard deviations of at least three independent experimental replicates (**A**), with the % repaired averages specified in (**B**).

To more extensively characterize the reliability and breadth of the assay, we performed time course analysis using three different human cell lines. In addition to the 293T embryonic kidney cells, we employed HeLa cervix epitheloid carcinoma and LN428 glioblastoma-derived cell lines. For the comparative assays, we used the PolyPlus INTERFERin transfection method, the standard MiniPrep lysis technique, and the streptavidin-capture strategy. Although it was found that BER typically peaks around 6–9 hr in HEK 293T cells (Fig. 4 and unpublished observations), HeLa and LN428 cells exhibited slower repair kinetics, with TG-BER peaking at ~12 hr or thereafter (Fig. S6). We chose 16 hr for the comparison, since BER was readily detectable at that time point in all three cell lines. As demonstrated in Fig. 5, BER was routinely observed in each of the three different cell types, with the recovered, repaired substrate ranging from ~3–8% at 16 hr post-transfection, and both cancer cell lines demonstrating lesser repair capacity than the HEK 293T cells.

#### Repair of TG in NTH1-deficient Cell Lines

**Figure 6:**
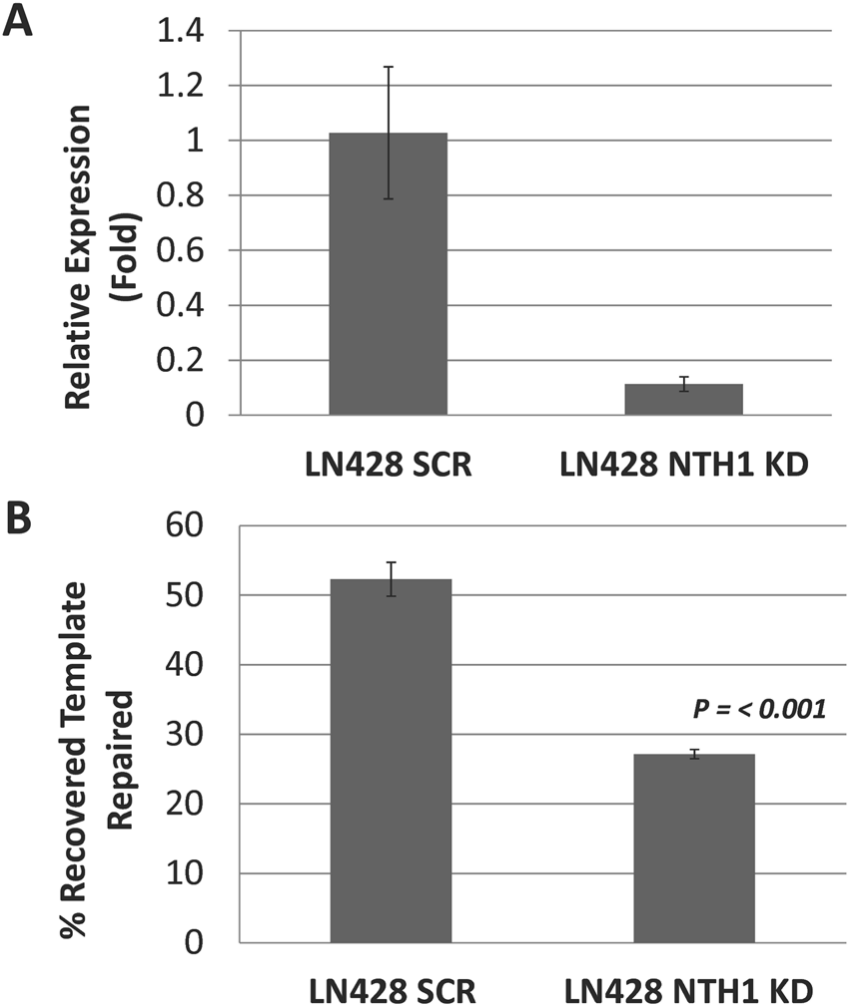
Dependence of TG Repair on NTH1 Status. (**A**) Efficiency of NTH1 knockdown (KD) relative to the scrambled (SCR) control. The *NTH1* mRNA levels of the SCR and NTH1 KD cells were determined using a Taqman Gene Expression Assay specific for human *NTH1* (Hs00267385_m1) and normalized to the expression of human β-actin (ThermoFisher Scientific). The results reported are averages +/− SE normalized to the SCR control (set as 1). (**B**) Reduced TG repair in NTH1 KD cells. Following transfection of the two-component TG substrate, a 6 hr incubation was carried out to compare the repair efficiency for TG in NTH1 KD and SCR control LN428 cell lines. Averages and standard deviations of percent-recovered template repaired from multiple experiments are shown. *P*-value < 0.001.

NTH1 is generally considered the major enzyme responsible for carrying out repair of TG lesions. To address this issue and test the validity of our assay further, we performed the TG ORA using an engineered human LN428 shRNA NTH1 stable knockdown (KD) cell line and its comparable scrambled (SCR) control; *NTH1* KD efficiency was determined to be >80% by qPCR (Fig. 6A). Following transfection of the TG two-component substrate and a 6 hr incubation, repair of the TG base lesion was quantified to be ~52% in the SCR control cells and ~27% in NTH1 KD cell line, revealing a marked impairment in the ability to resolve TG lesions in the absence of the NTH1 glycosylase (Fig. 6B).

#### Comparative Analysis of TG and TT-dimer

**Figure 7:**
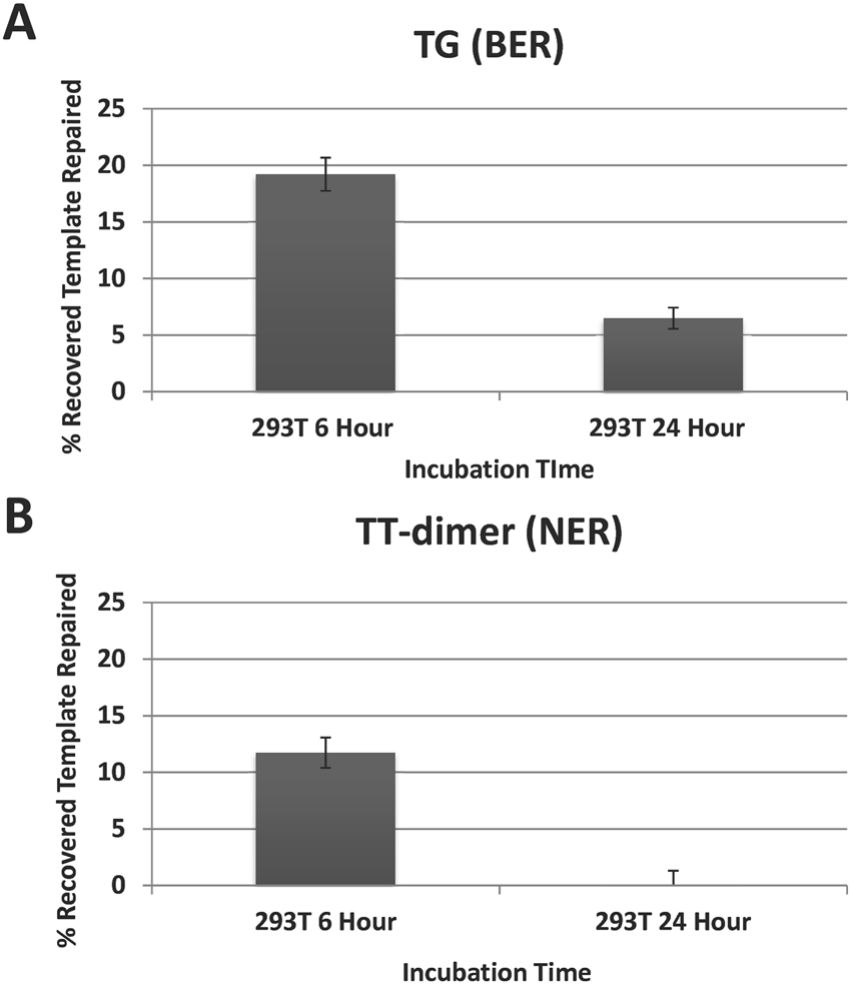
Comparison of TT-dimer (NER) and TG (BER) ORA. Two time-points, 6 and 24 hr, were utilized as points of comparison between a TG-containing substrate (**A**) and a TT-dimer-containing substrate (**B**) transfected into and recovered from HEK 293T cells using our established protocol. Averages and standard deviations of percent-recovered template repaired are shown.

In an attempt to determine the utility of the BER version of the ORA substrate relative to a comparable TT-dimer substrate designed for NER, we performed simultaneous analysis of the two substrates in HEK 293T cells (Fig. 7). Using the optimized protocol, repair measurements were made for each substrate following transfection, lysis and recovery at 6 hr and 24 hr time points; chosen because our previous experiments suggested that the TG-containing substrate was stable for up to 24 hr, with repair peaking around 6 hr, and because maximum repair of the TT-dimer substrate was reported to occur at 24 hr in 293T cells^26^. For the TG-containing substrate, we observed, on average, ~19% and 6% repair of the recovered substrate at 6 and 24 hr, respectively (Fig. 7A). For the TT-dimer, we observed, on average, 12% and 0% repair at 6 and 24 hr, respectively (Fig. 7B). At 24 hr, there was a greater range of biological variability observed between replicates, particularly when employing the TT-dimer substrate. These results utilizing the TT-dimer substrate in HEK 293T cells are contrary to those reported previously^28^, and may have arisen for a number of reasons. First, our newly designed 2-component substrate is different than the originally described 3-component hairpin. Second, there are differences between the nucleotide sequences utilized and G:C content, which may affect substrate stability over time. Third, the transfection and lysis techniques optimized by our group are different from those utilized previously. Nevertheless, we found that in the context of HEK 293T cells, both substrates were competent for repair measurements at 6 hr.

## Discussion

We describe herein the development of a cell-based assay that measures whole-pathway BER capacity in human cells, adapting and optimizing the basic ORA methodology previously described to detect NER^28^. Although variability was observed among the different cell lines analyzed, the TG-BER ORA was able to reliably detect repair in three different human cell types: HEK 293T, HeLa and LN428. Presumably this variability stems from: (i) intrinsic differences in cell phenotypes; (ii) differences in intracellular substrate localization or stability; (iii) differences in BER rates; and (iv) differences in methodological performance. For example, when assays were conducted in HEK 293T cells on the same day, repair kinetics appeared to be fairly uniform, with ΔCt_Test_ variations of ~1–2 cycles. However, repair kinetics appeared to change bi-directionally with even a few subsequent passages of the same cell line, sometimes resulting in ΔCt_Test_ variations of ~2–5 cycles. HeLa and LN428 cell lines appeared to be less prone to biological variability independent of passage number. Since both HeLa and LN428 are cancer cell lines, which are not prone to senescence in cell culture, this phenotype may at least partially explain the observed variability. Our experience has taught us that such issues represent some of the limitations of the assay, and thus, for those wishing to implement the BER ORA, careful attention should be paid to standardizing the assay protocol across all steps, including timing and passage number, as well as the transfection, lysis and recovery techniques for your particular cell type.

Our work here focused on the base lesion TG, since it demonstrated an effective blocking potential in the qPCR and requires repair to be initiated by a DNA glycosylase (specifically, NTH1), thus engaging each step of classic BER. The abasic site (F), though a more potent block of the qPCR polymerase, would not allow for a comprehensive measurement of whole-pathway BER (since it lacks the glycosylase step), and is a synthetic analog that requires long-patch BER for resolution. Nevertheless, using a two-component version of the F substrate, we found that the lesion was a potent block to the qPCR polymerase, effectively inhibiting the reaction, indicating a potential utility of F in the context of the assay. Notably, preliminary analysis revealed ~65% repair of the recovered template following transfection of the F-substrate into LN428 cells and incubation for 8 hr (unpublished observation), implying that the ORA could potentially be used to assess long-patch BER.

While additional aspects of the BER ORA can undoubtedly be improved, a key emphasis going forward will be adapting the method for use with non-blocking lesions. To examine the possibility of exposing a non-blocking substrate to a repair enzyme and converting it to a PCR polymerase block, we treated our three-component 8oxoG substrate with (versus without) Fpg, an *Escherichia coli* DNA glycosylase that excises 8oxoG and coverts the AP site product to a DNA strand break^1^. We observed that untreated 8oxoG substrate efficiently amplified the Test region, whereas the Fpg-treated 8oxoG DNA was no longer able to effectively amplify across the lesion site; undamaged control DNA was unaffected by Fpg exposure (Fig. S7). Thus, going forward, these results suggest that recovered material following transfection and repair incubation could be treated prior to qPCR to get a measure of the amount of repaired substrate (i.e., in the case here, unresponsive to Fpg treatment), relative to an established “repair curve”. In addition, as noted earlier, it may be valuable to examine qPCR Test primer positioning with the other base damages, or one could consider taking advantage of the mutagenic properties of the modifications. For example, if 8oxoG was used in the initial substrate and went unrepaired, the Test PCR products could be sequenced and analyzed for normal G:C content (repaired) versus T:A, which would reflect the presence of 8oxoG, as A is typically inserted opposite this base lesion^33^. Thus, RNAseq of the PCR products obtained on the recovered substrate/product could be used as an indirect means of measuring BER capacity.

There has been some debate concerning the role of BER, specifically NTH1, in the resolution of TG^1,2,28^. For example, a previous study found that endogenous modified bases in genomic DNA from *neil1* ^*−/−*^ *nth1* ^*−/−*^ double knockout mice did not exhibit a significant increase in the levels of oxidized pyrimidines when compared to wild-type or single mutant mice^34^, suggesting an alternative repair mechanism that may involve the NEIL1/PNKP sub-pathway. In addition, some have argued a role for NER in TG removal^1,28^. Our studies using NTH1 KD and SCR control cells not only validate our BER ORA, but support a prominent role for the NTH1 glycosylase in the repair of TG in DNA.

In addition to cell lines, a long-term interest is in applying the ORA to primary cell material in the context of defined epidemiology studies. Peripheral blood mononuclear cells (PBMCs) are commonly used in population studies since they are readily accessible and cost-effective for large-scale investigations. However, this cell mixture, consisting of lymphocytes, monocytes and other cell types, is seemingly suboptimal for repair capacity measurements using the ORA, as efficient introduction of non-viral-based vectors is difficult to achieve. Indeed, our preliminary work indicates a marginal ability of the ORA to measure TG-directed BER in freshly isolated PBMCs (unpublished observation), suggesting that work is needed to optimize the methods of cell stimulation (i.e., phytohemagglutinin activation) and transfection to reliably measure repair. Given the issues associated with PBMCs, primary fibroblasts, for example, may be a more suitable target cell type, as they are amenable to lipid and peptide-based transfection techniques, without the need for further manipulation in culture.

The known factors affecting repair kinetics within a given cell or tissue type are numerous, and include the intracellular environment, cell metabolism, the initiating lesion, lesion location, sequence context, accessibility to repair factors, polymerase and helicase-blocking potential of the lesion, as well as additional exogenous factors. In order to account for these elements, large-scale, rigorously-controlled studies must be carried out that are capable of measuring repair, ideally with multiple robust techniques. The assay described herein represents a valuable tool towards holistically measuring DNA repair capacity, and may complement existing strategies to develop a clearer picture of the complex nature of and pathway coordination required for DNA repair. We believe that with context-dependent optimization, the BER ORA could be adapted to answer many pertinent questions going forward.

## Materials and Methods

### Human Cells and Reagents

HEK 293T cells and HeLa cervix epitheloid carcinoma cells were obtained from ATCC (Cat. No. CRL-11268 and CCL-2, respectively). OptiMEM serum-free medium, for use with transfection, was obtained from ThermoFisher Scientific. PolyPlus INTERFERin was obtained from VWR. Oligonucleotides were synthesized by Midland and IDT. HeLa and 293T cells were maintained in DMEM (ThermoFisher) supplemented with 10% FBS under antibiotic-free conditions. Generally, the HEK 293T cells used in the experiments were between passage 6 and 10 post ATCC source culture, and were split 1:8 at each passage. Molecular biology enzymes were purchased from New England Biolabs. Cell lysis solutions (P1 thru P3) were obtained from Qiagen. Magnetic streptavidin beads were from ThermoFisher.

### NTH1 KD and SCR Control Cell Lines

Cells depleted of NTH1 (NTH1 KD) were developed by transduction of LN428 cells with lentivirus expressing NTH1-specific shRNA, essentially as described^35^. Briefly, lentiviral vectors expressing a shRNA specific to NTH1 (NM_002528.4-844s1c1, GCACGAGATCAATGGACTCTT) or the corresponding scrambled shRNA (SCR) control were obtained from Sigma and prepared by the MCI Gene Expression, Engineering and Discovery (GEED) Facility at the University of South Alabama. Lentiviral particles were generated by co-transfection of 4 plasmids [SCR or NTH1-specific shRNA plasmid (pLK0-Puro) with pMD2.g(VSVG), pVSV-REV and pMDLg/pRRE] into 293FT cells using TransIT-X2® Dynamic Delivery System (Mirus Bio LLC). LN428 cells (1 × 10^4^) were seeded into a 6-well plate 24 hr before transduction, and then transduced twice with the same shRNA lentiviruses with an 18 hr incubation at 32 °C followed by an 8 hr incubation at 37 °C. Cells were selected in growth media containing puromycin (1.0 μg/ml) for two weeks. Validation of NTH1 KD was performed by quantitative reverse transcription-PCR (qRT-PCR) using an Applied Biosystems StepOnePlus system via the ΔΔCT method. cDNA was prepared using the Taqman Gene Expression Cells-to-CT kit (ThermoFisher Scientific) according to the manufacturer’s instructions.

### DNA Substrate Assembly

For the three-component substrate, the relevant oligonucleotides (Table 1) were mixed at 10 µM each and annealed by heating to 90 °C and cooling slowly (water bath or PCR cycler). DNAs were mixed with 1x T4 DNA ligase buffer supplemented with fresh 1 mM ATP and 10 mM DTT, and 100 units T4 polynucleotide kinase. Following a 2 hr incubation at 37 °C, 400 units of T4 DNA ligase were added, along with an additional 1 mM ATP and 10 mM DTT. This reaction mix was incubated overnight at room temperature and analyzed on a polyacrylamide gel and used in subsequent repair experiments. For the two-component substrate, each oligonucleotide (Table 1) was combined at 10 µM each, and incubated overnight at room temperature. The kinase step was omitted, since the “hairpin” oligonucleotide was 5′-phosphorylated during production, and the ligase reaction was performed as above.

### Cell Transfection

For HEK 293T cells, which were maintained in DMEM medium supplemented with FBS to 10% at 37 °C and 5% CO_2_, transfection was typically performed in a 6-well tissue culture plate at 60–80% confluency. PolyPlus INTERFERin, selected as our preferred method based on experimental comparisons, was used per manufacturer’s instructions. For Lipofectamine 2000 (ThermoFisher), a complex of DNA and Lipofectamine was established by combining (a) 2 µL of Lipofectamine in 50 µL of Optimem serum-free medium and (b) 100 pM of the prepared substrate in 50 µL of Optimem serum-free medium. This mixture (total volume of 100 µL, for each transfection) was incubated for 10 min at room temperature and then added to each well containing fresh medium and the 293T cells. Cells were incubated with the transfection cocktail for the duration of the experiment (time dependent on when repair was assessed) with no medium replacement. Other lipid transfection methods (Xtreme Gene HP from Roche, Lipofectamine RNAiMax from ThermoFisher) and nucleofection methods (Amaxa from Lonza) were used per manufacturer’s instructions.

### DNA Recovery

To recover DNA from adherent cells, cells were washed twice with 1x PBS in their respective wells. Cells were then lysed in a 6-well plate, by resuspension in 500 µL Qiagen buffer P1 with RNase. A 500 µL volume of Qiagen buffer P2 was added, combined gently by mixing, and allowed to stand for 5 min at room temperature. A 500 µL volume of Qiagen buffer P3 was then added, and mixed thoroughly. Following a brief incubation, the solution was transferred to an Eppendorf tube and the precipitant removed by high speed centrifugation (at least 14000 rcf) for 3 min. The supernatant was retained, passed through a 0.45 µm filter, and subsequently processed to purify hairpin DNA. In situations involving poorly adherent cells (e.g. 293T), cells were mechanically harvested from 6-well plates by centrifugation at 1000 rfc for 5 min prior to washing, lysis and supernatant recovery as above.

The hairpin-containing supernatant was diluted in 10 mL of water in a 15 mL conical tube. To isolate the 5′-biotinylated hairpin DNA using magnetic streptavidin beads, the beads were prepared by dissolving 10 µl (per experimental sample) in ~1 ml of sterile water. Beads were collected by centrifugation at 1000 rfc, and washed twice with water, before incubating in water for 1 hr. Beads were harvested one final time, and resuspended in their initial starting volume. Ten µL of this suspension was then added to each 10 mL hairpin-containing solution (see above), and incubated for 15 to 45 min while tumbling. The beads were subsequently captured using a QIAGEN 12-tube magnetic stand by incubating 1 mL of each sample in an Eppendorf tube against the magnet for 5 min at room temperature. After removing the solution by careful pipetting, beads were washed with 1 ml of each 1X TE buffer, water and ethanol (in that order) by resuspending them and capturing them as described above, each time removing the supernatant. After the washing steps, 20 µL of ethanol was added to the side of the tube to help capture any remaining beads at the bottom of the tube. The ethanol was then allowed to evaporate, and the bead pellet was resuspended in 20 µL of water, transferred to a PCR tube, and heated to 80 °C for 1 hr to denature the streptavidin and free the biotinylated molecules. The beads were subsequently pelleted by centrifugation at ~1000 rfc for 2 min, and the supernatant was retained for subsequent qPCR.

Where indicated, hairpin DNA was isolated using a buffer exchange column, such as the Amicon Ultra 10 kD molecular weight cutoff (EMD Millipore). In brief, the hairpin-containing supernatant (see above) was added to the column and centrifuged at 14000 rcf for 15 min (repeated as necessary) to reduce the volume of solution to 30 µL; the hairpin is retained in the exclusion portion because its molecular weight is ~56 kD. The 30 µL solution was diluted again to 400 µL with water, and centrifuged as above to reduce it to an appropriate volume (between 20 and 100 µL depending on the hairpin yield and desired number of PCRs). The final hairpin-containing solution was then collected by spinning the filter column upside-down for 2 min at 1000 rcf, and aliquots of this material were used accordingly in qPCR.

### qPCR

For qPCR, two separate reactions were prepared for the Reference and Test amplifications. In short, reaction cocktails consisted of 10 µL SYBR Power 2x qPCR Master Mix (ThermoFisher), which contains the polymerase, the recovered hairpin DNA (typically 1 µL, but <10% the final volume of the reaction), 200 nm final concentration of each primer (see Table 2 and figure legend), and ultrapure water (EMD Millipore) to a final volume of 10–20 µL. We note that the resolving range of the method is somewhere between 1 femtomole and 1 attomole of template, since fewer molecules result in Test primer false positives, and more molecules meant Reference primer amplification before heat touchdown was finished. Reaction items were mixed well before subjecting to the following cycling conditions: 10 min, 95 °C (hot start); 10 cycles of 15 sec, 95 °C and 60 sec, 64 °C (temperature decreasing at 0.4/cycle); 30 cycles of 15 sec, 95 °C and 60 sec, 60 °C.

## Electronic supplementary material

Supplementary Figures
