# Supplementary material for: Development of a Cell-Based Assay for Measuring Base Excision Repair Responses
Source: Sci Rep. 2017 Oct 11;7:13007. doi: 10.1038/s41598-017-12963-7 (PMC5636817; doi:10.1038/s41598-017-12963-7)
Supplement: Supplementary file 1 — Supplementary Figures [file 41598_2017_12963_MOESM1_ESM.pdf]

Development of a Cell-Based Assay for Measuring Base Excision Repair Responses

Tyler Golato[1]#, Boris Brenerman[1]#, Daniel R. McNeill[1], Jianfeng Li[2], Robert W. Sobol[2], David M. Wilson III[1]*

[1]Laboratory of Molecular Gerontology, National Institute on Aging, Intramural Research Program, National Institutes of Health, 251 Bayview Blvd., Ste. 100, Baltimore, MD 21224 USA
[2]Molecular and Metabolic Oncology Program, USA Mitchell Cancer Institute, University of South Alabama, 1660 Springhill Avenue, Mobile, AL 36604 USA

#Authors contributed equally.

*To whom correspondence should be addressed.

Tel: +1-410-558-8153; Fax: +1-410-558-8157; E-mail: wilsonda@mail.nih.gov

qPCR Profiles for BER Lesions

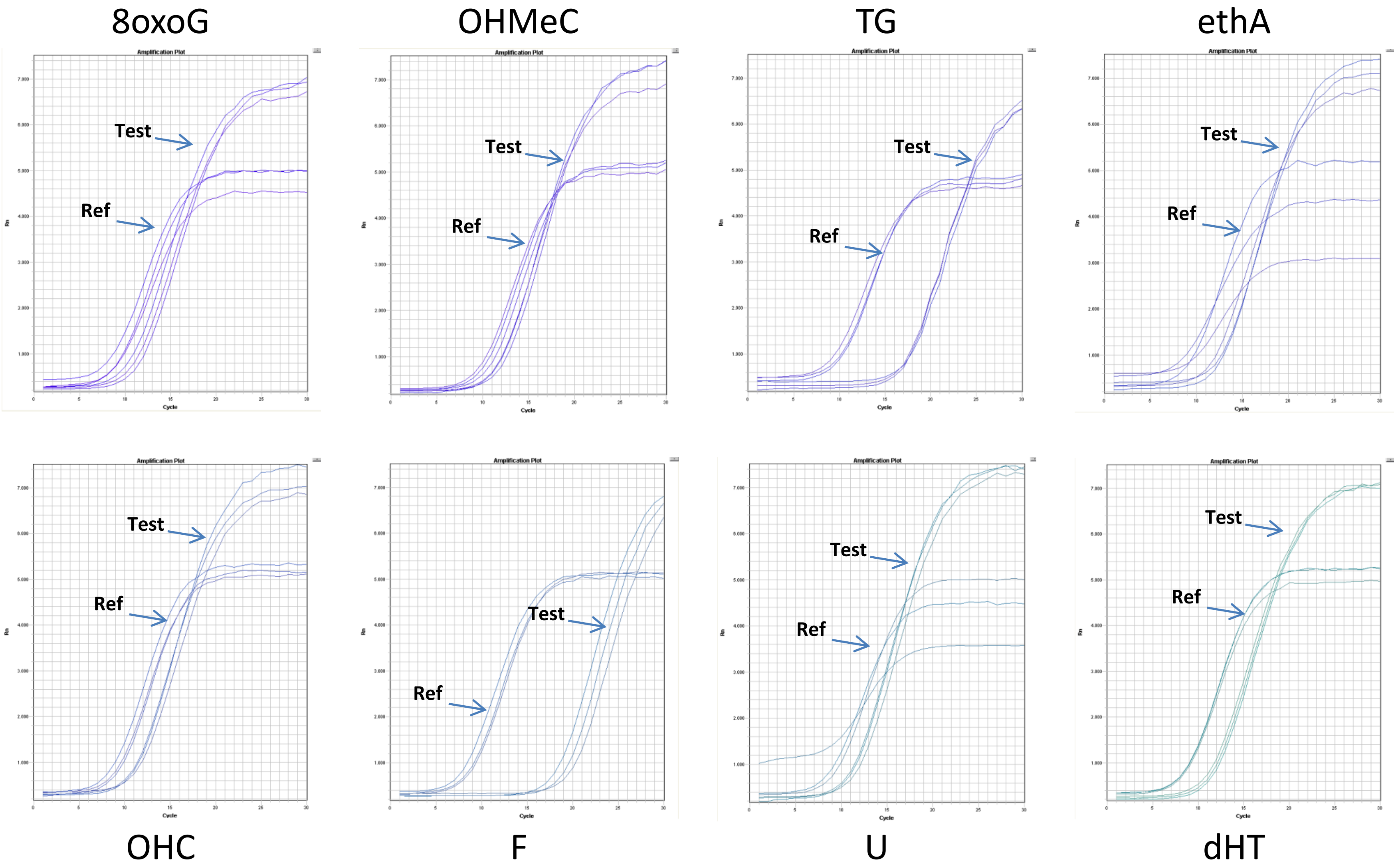

Figure S1

Figure S1. **qPCR Amplification Profiles for Classic BER Lesions.** Amplification curves are displayed and correspond to a 3-component substrate containing one of the following lesions: 8oxoG, OHMeC, TG, ethA, OHC, F, U or dHT. The distance between the “Test” and “Ref” (reference) qPCR curves is correlated to the sensitivity of the assay (ΔCt), with greater separation indicating greater sensitivity (or blocking power). Only TG and F lesions yielded curve separation appropriate for utilization as the primary substrate. Y-axis is fluorescent intensity of qPCR; X-axis is cycle number.

Condition optimization results

A

Conventional amplification

Optimized primer position

B

LNA + Heat Touchdown

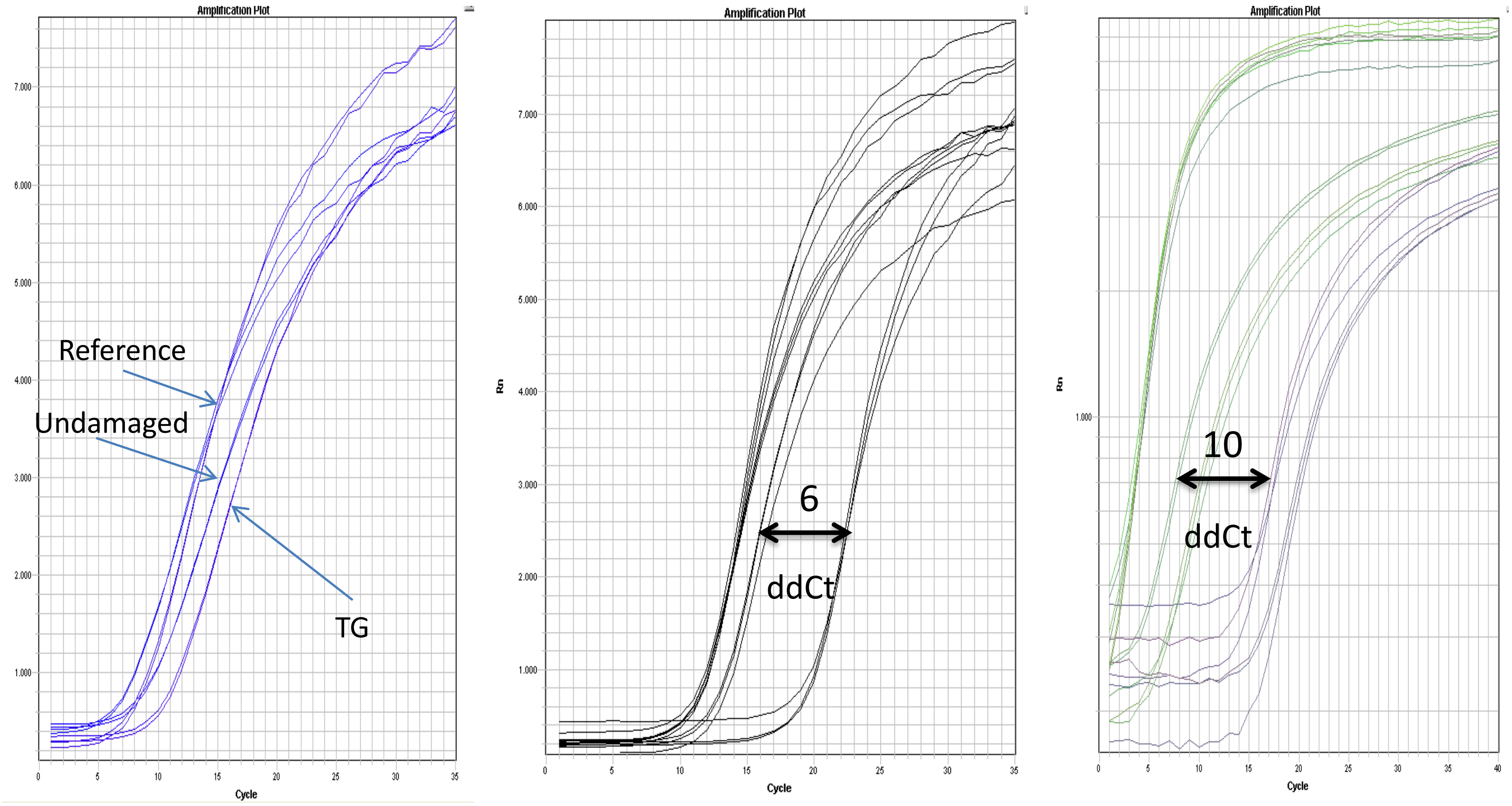

Figure S2

Figure S2. **PCR Condition Optimization Results.** (A) Conventional amplification (left), in comparison to the optimized primer position, yielded poor curve separation of test for undamaged versus TG templates. Reference amplification was similar for both substrates. Once primer position was optimized (right), ΔΔCt was improved to >6 cycles, vastly improving the sensitivity of the assay. (B) LNAs, in addition to a heat touchdown step, further improved assay sensitivity by increasing ΔΔCt to >10 cycles. The heat touchdown step was maintained, but LNAs were not utilized during further assay development, as overall amplification efficiency was lost. See Figure S1 for further details.

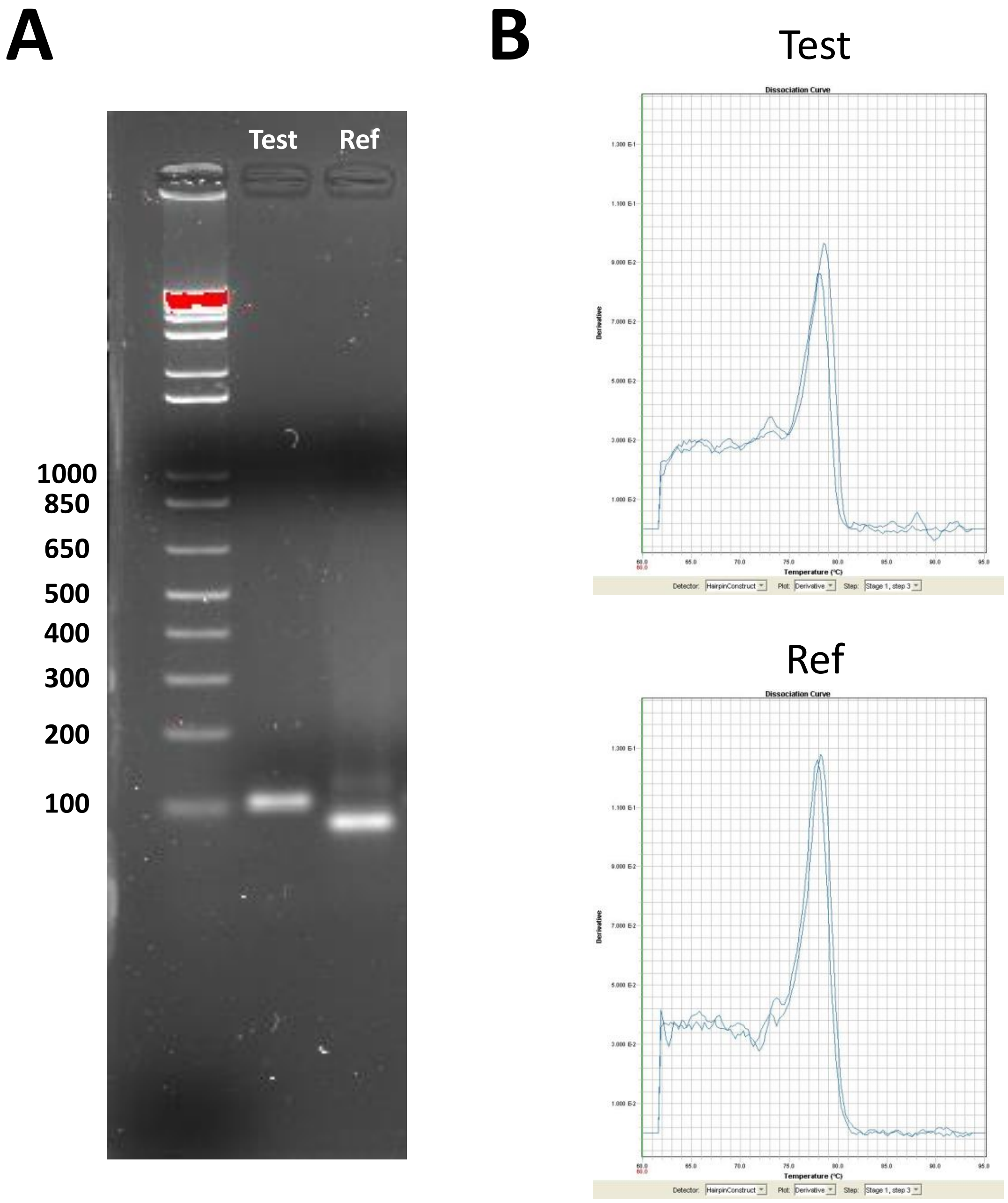
A
B
Test
Ref
1000
850
650
500
400
300
200
100
Test
Dissociation Curve
Derivative
Temperature (°C)
Ref
Dissociation Curve
Derivative
Temperature (°C)

Figure S3

Figure S3. **qPCR Product Analysis.** (A) Agarose Gel. qPCR was performed on the Test and Ref (reference) regions of the 2-component control template, and the PCR product was subsequently run on a 2% agarose gel stained with ethidium bromide. Expected size is 90 bp for Test and 57 bp for Ref. Left lane shows the 1k Plus DNA Ladder (ThermoFisher Scientific), and specific bp fragments, as a reference. (B) Melting Curve. Melting curves were generated on the qPCR products from the Test or Ref amplification using the undamaged control hairpin substrate. Curves, presented in triplicate, indicate clean reaction conditions with product indicated by large peak. Curve is generated as a function of the derivative (y-axis) over the temperature (x-axis).

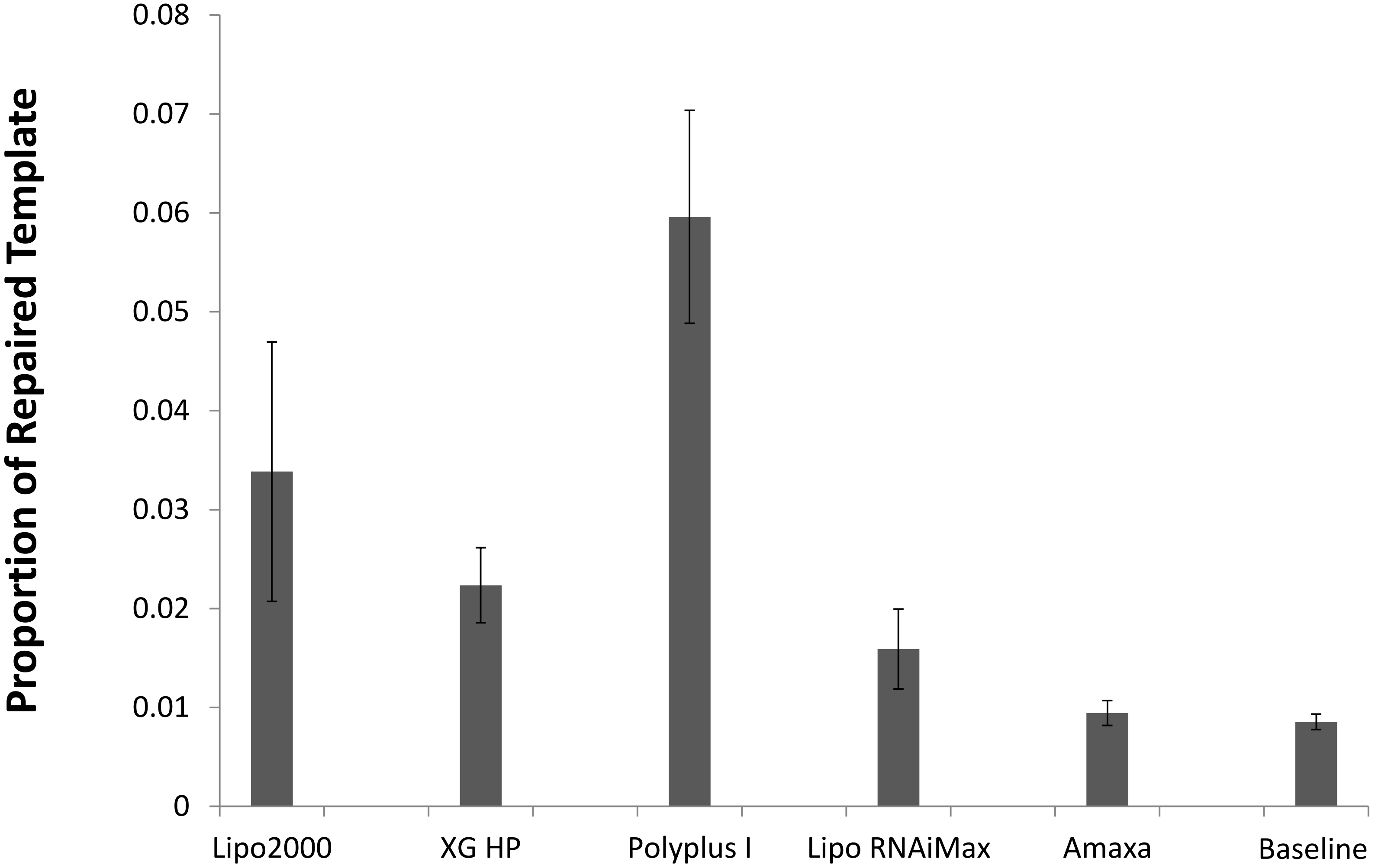
Proportion of Repaired Template
0.08
0.07
0.06
0.05
0.04
0.03
0.02
0.01
0
Lipo2000
XG HP
Polyplus I
Lipo RNAiMax
Amaxa
Baseline

Figure S4

Figure S4. **Comparison of Transfection Methods.** HEK 293T cells were transfected using the indicated transfection strategy, and repair was allowed to proceed for 16 hr. Cells were lysed normally, and hairpin DNA collected by streptavidin-capture and analyzed by qPCR. The averages and standard deviations of three data points representing the proportion of repaired to unrepaired template are shown for each method.

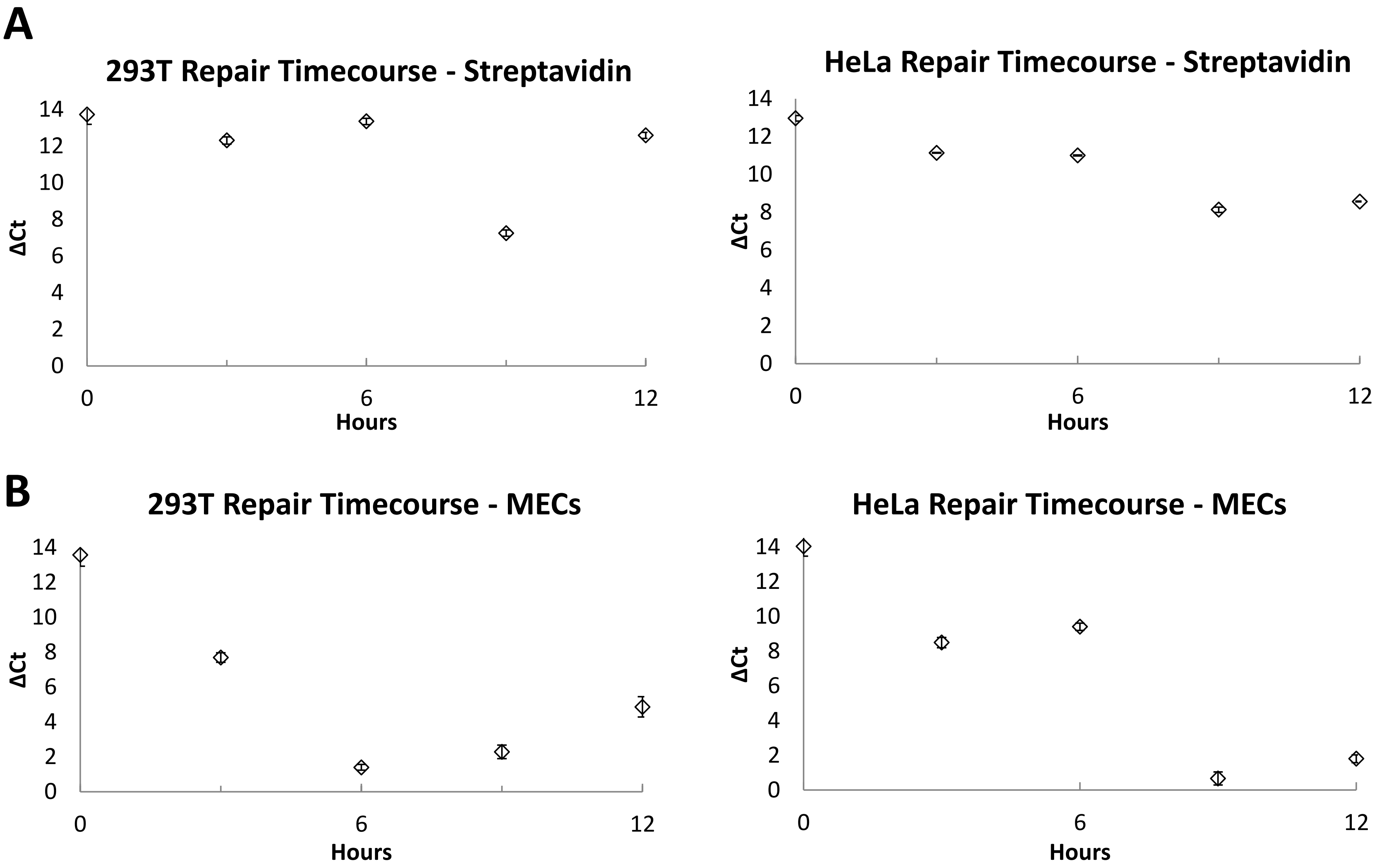
A
293T Repair Timecourse - Streptavidin
HeLa Repair Timecourse - Streptavidin
ΔCt
Hours
0
6
12
0
2
4
6
8
10
12
14
B
293T Repair Timecourse - MECs
HeLa Repair Timecourse - MECs
ΔCt
Hours

Figure S5

Figure S5. **Recovery Time-course in HEK 293T and HeLa Cells with Streptavidin-Beads or Molecular Exclusion Columns.** Depiction of a time-dependent decline in ΔCt_{Test} (inversely correlated to double-strand product formation and amplification efficiency) in 293T or HeLa cells after transfection with PolyPlus INTERFERIN, purified by either (A) streptavidin-capture or (B) molecular exclusion filtration columns (MECs). Plotted are the averages and standard deviations of at least three independent experimental replicates.

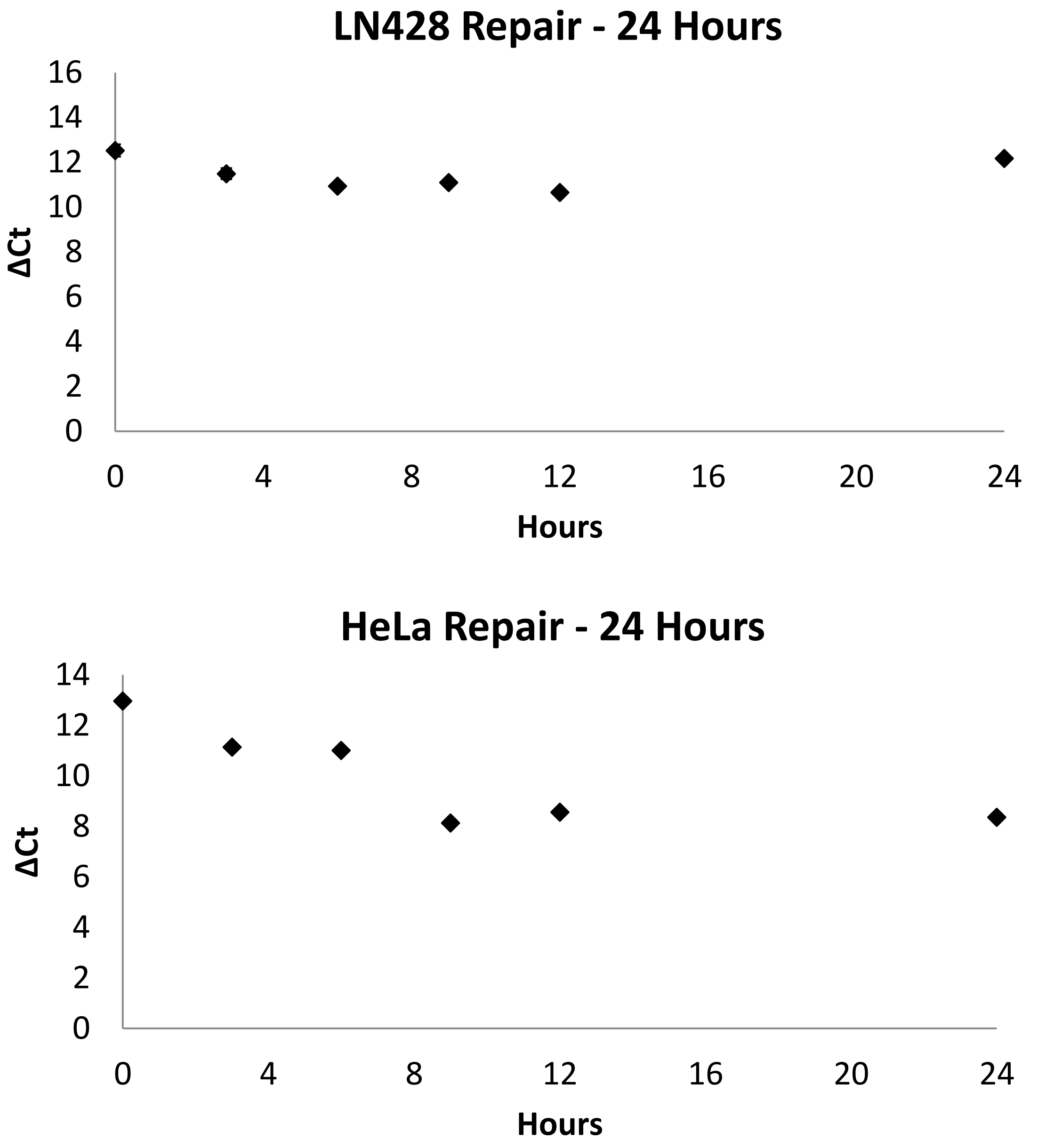

LN428 Repair - 24 Hours
ΔCt
Hours
0 2 4 6 8 10 12 14 16
0 4 8 12 16 20 24
HeLa Repair - 24 Hours
ΔCt
Hours
0 2 4 6 8 10 12 14
0 4 8 12 16 20 24

Figure S6

Figure S6. **24 Hour Repair Time-course in LN428 and HeLa.** Time-dependent decrease in ΔCt indicates repair taking place over time in LN428 and HeLa cell lines. Repair appears to peak between 12-24 hours, indicating slower repair kinetics than HEK 293T.

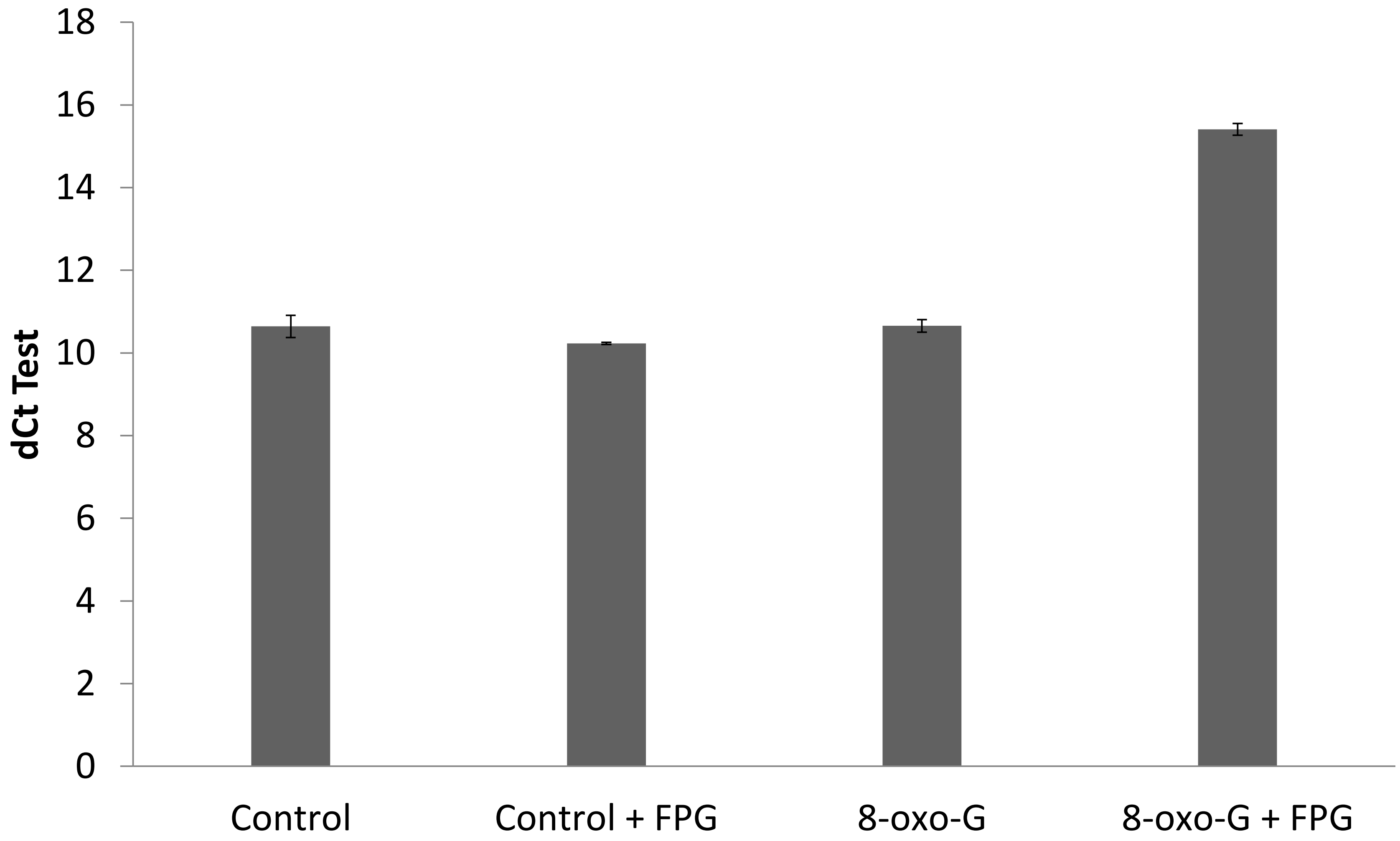
18
16
14
12
10
8
6
4
2
0
dCt Test
Control
Control + FPG
8-oxo-G
8-oxo-G + FPG

Figure S7

Figure S7. **qPCR Test Product Amplification Comparison in Fpg-treated/untreated Control and 8oxoG-containing Substrates.** Control and 8oxoG-containing 3-component substrates were PCR amplified with or without pre-treatment with Fpg glycosylase (NEB), which generates an AP site or strand break capable of blocking the qPCR polymerase. Results for amplification of the test region indicate the generation of a block with the 8oxoG-containing substrate following treatment with Fpg, suggesting that non-blocking substrates can be analyzed with the ORA with the addition of a DNA repair (e.g. glycosylase) digestion step after retrieval.
